# MRI-MECH: mechanics-informed MRI to estimate esophageal health

**DOI:** 10.3389/fphys.2023.1195067

**Published:** 2023-06-09

**Authors:** Sourav Halder, Ethan M. Johnson, Jun Yamasaki, Peter J. Kahrilas, Michael Markl, John E. Pandolfino, Neelesh A. Patankar

**Affiliations:** ^1^ Theoretical and Applied Mechanics Program, McCormick School of Engineering, Northwestern University, Evanston, IL, United States; ^2^ Department of Radiology, Feinberg School of Medicine, Northwestern University, Chicago, IL, United States; ^3^ Department of Mechanical Engineering, McCormick School of Engineering, Northwestern University, Evanston, IL, United States; ^4^ Department of Medicine, Feinberg School of Medicine, Division of Gastroenterology and Hepatology, Northwestern University, Chicago, IL, United States

**Keywords:** MRI, esophagus, physics-informed neural network, computational fluid dynamics, deep learning, lower esophageal sphincter, active relaxation, esophageal wall properties

## Abstract

Dynamic magnetic resonance imaging (MRI) is a popular medical imaging technique that generates image sequences of the flow of a contrast material inside tissues and organs. However, its application to imaging bolus movement through the esophagus has only been demonstrated in few feasibility studies and is relatively unexplored. In this work, we present a computational framework called mechanics-informed MRI (MRI-MECH) that enhances that capability, thereby increasing the applicability of dynamic MRI for diagnosing esophageal disorders. Pineapple juice was used as the swallowed contrast material for the dynamic MRI, and the MRI image sequence was used as input to the MRI-MECH. The MRI-MECH modeled the esophagus as a flexible one-dimensional tube, and the elastic tube walls followed a linear tube law. Flow through the esophagus was governed by one-dimensional mass and momentum conservation equations. These equations were solved using a physics-informed neural network. The physics-informed neural network minimized the difference between the measurements from the MRI and model predictions and ensured that the physics of the fluid flow problem was always followed. MRI-MECH calculated the fluid velocity and pressure during esophageal transit and estimated the mechanical health of the esophagus by calculating wall stiffness and active relaxation. Additionally, MRI-MECH predicted missing information about the lower esophageal sphincter during the emptying process, demonstrating its applicability to scenarios with missing data or poor image resolution. In addition to potentially improving clinical decisions based on quantitative estimates of the mechanical health of the esophagus, MRI-MECH can also be adapted for application to other medical imaging modalities to enhance their functionality.

## 1 Introduction

The esophagus plays a crucial role in the functioning of the gastrointestinal tract, and esophageal disorders are associated with reduced quality of life. There is a high worldwide prevalence of esophageal disorders, as exemplified by studies (El-Serag et al., 2014; Yamasaki et al., 2018) reporting that gastroesophageal reflux disease (GERD) has a prevalence of 18.1%−27.8% in North America alone, with an increase across all age groups. Another study (Bhattacharyya, 2014) reported that dysphagia (swallowing difficulty) affects 1 in 25 adults annually in the United States. Hence, it is important to improve current diagnostic technologies for esophageal disorders. Some of the common tests for diagnosing esophageal disorders are barium esophagram using fluoroscopy, high-resolution manometry (HRM) (Fox et al., 2004; Pandolfino et al., 2007; Fox and Bredenoord, 2008; Pandolfino et al., 2008; Pandolfino et al., 2009), and functional luminal imaging probe (FLIP) (Gyawali et al., 2013; Carlson et al., 2016). An esophagram is a non-invasive test wherein a patient swallows a radiopaque material, usually dilute barium, and fluoroscopic imaging is used to visualize the esophageal lumen. HRM and FLIP are more invasive procedures where a catheter with sensors is inserted into the esophagus to quantitatively assess the esophageal contractility. Measurements made by HRM and FLIP are physical quantities such as the pressure developed within the esophagus when a fluid is swallowed and/or the cross-sectional area variation along the esophageal length. Variations in these physical quantities are the consequence of more fundamental esophageal physiomarkers, such as the stiffness of the esophageal walls, active contraction of the esophageal musculature, and active relaxation. However, clinical decisions are made based on the qualitative or quantitative patterns of these physical quantities rather than the physiomarkers that cause them. For example, the widely used Chicago Classification v4.0 (CCv4.0) (Yadlapati et al., 2021) classifies esophageal disorders based on a set of parameters derived from pressure measurements made with HRM. The explanation for this is that it is difficult to measure the fundamental physiomarkers, which occur at molecular, cellular, and tissue levels. Since luminal pressure and cross-sectional area, which occur at the tissue level, are the physical quantities commonly measured by HRM and FLIP, the first stage of quantifying the fundamental physiomarkers of esophageal function is at the tissue level. In this context, the mechanical properties of the esophageal wall and its dynamic behavior related to active contraction and relaxation could be important physiomarkers. Thus, a mechanics-based analysis may provide valuable mechanistic insights regarding esophageal function.

Previous mechanics-based studies on the esophagus have been conducted both experimentally and computationally. Experimental studies (Fan et al., 2004; Yang et al., 2006; Natali et al., 2009; Stavropoulou et al., 2009; Sokolis, 2013) focused on the mechanical properties of the esophageal walls *in vitro*. *In silico* modeling of the esophagus has been performed both in the context of pure fluid mechanics (Brasseur, 1987; Li and Brasseur, 1993; Li et al., 1994; Ghosh et al., 2005; Acharya et al., 2021a) to understand the nature of bolus transport and in fully resolved fluid-structure interaction models to understand how the esophageal muscle architecture influences esophageal transport and the stresses developed in the esophageal walls during bolus transport (Kou et al., 2015; Kou et al., 2017; Halder et al., 2022a). A systematic review of the various constitutive models of the esophagus and the other organs of the gastrointestinal tract was conducted by Patel et al. (2022). *In silico* mechanics-based analyses (Acharya et al., 2021b; Halder et al., 2021; Halder et al., 2022b) have also been performed on data obtained from various diagnostic devices to identify mechanics-based physiomarkers. Acharya et al. (2021b) used a mechanics-based approach to calculate the work carried out by the esophagus in opening the esophagogastric junction (EGJ) and the necessary work required to open the EGJ using data obtained from FLIP. Halder et al. (2021) introduced a framework called FluoroMech applied to fluoroscopy images to estimate the mechanical health of the esophagus through quantitative estimates of esophageal wall stiffness and active relaxation. FluoroMech enhances the capability of fluoroscopy by adding quantitative predictions to fluoroscopy data, which are inherently qualitative in nature. In this work, we present a framework called MRI-MECH, which uses dynamic MRI as input to estimate esophageal health through mechanics-based metrics for active relaxation and wall stiffness.

Both FluoroMech and MRI-MECH utilize the input of esophageal cross-sectional area, which varies as a function of time and length along the esophagus. However, there are some key differences in their approach that can be classified into two categories. The first category pertains to the differences between fluoroscopy and dynamic MRI. Fluoroscopy is an older and simpler approach wherein X-ray imaging is used to visualize a swallowed bolus passing through the esophagus, resulting in a video with high temporal resolution but only a two-dimensional projection of the bolus. Hence, the three-dimensional geometry of the bolus is unknown. Fluoroscopy is a well-established clinical test. Dynamic MR imaging, on the other hand, is a relatively complicated and evolving technology. MRI has been used to detect esophageal cancers (Petrillo et al., 1990; Riddell et al., 2006), but dynamic MRI has been used only in limited feasibility studies (Panebianco et al., 2006; Kulinna-Cosentini et al., 2007; Marciani, 2011). Most of these studies visualize esophageal wall movement through 2D imaging sequences using orally administered Gd contrast. In their current state, dynamic MRI images have a significantly lower temporal resolution but a very detailed three-dimensional representation of the bolus. However, dynamic MR imaging is currently not a standard practice for evaluating esophageal disorders, offering a vast potential for improvement. The second category of differences between FluoroMech and MRI-MECH lies in the implementations of the frameworks. FluoroMech uses the finite volume method to predict esophageal wall stiffness and active relaxation with the variation of the cross-sectional area as input. It is computationally fast (less than a minute) and requires very limited computational resources, but it requires a complete dataset of the variation of the cross-sectional area. Assumptions are required regarding the 3D shape of the bolus based on the volume of fluid swallowed, and since model predictions are sensitive to cross-sectional area variation, inaccuracies in measurements reflect on the predictions as well. MRI-MECH, on the other hand, uses a physics-informed neural network (PINN) (Raissi et al., 2019) to make predictions and is computationally demanding (takes approximately 1 hour to run), requiring better hardware, especially the GPU, to train the PINN. However, MRI-MECH is not sensitive to missing or imperfect measurements. Additionally, it does not require assumptions regarding the esophageal lumen cross-sectional shape because MRI provides three-dimensional geometry of the esophageal lumen. In the following sections, we describe the MRI-MECH framework in detail, along with its application to a dynamic MRI sequence.

## 2 Materials and methods

### 2.1 Accelerated dynamic MRI

**FIGURE 1:**
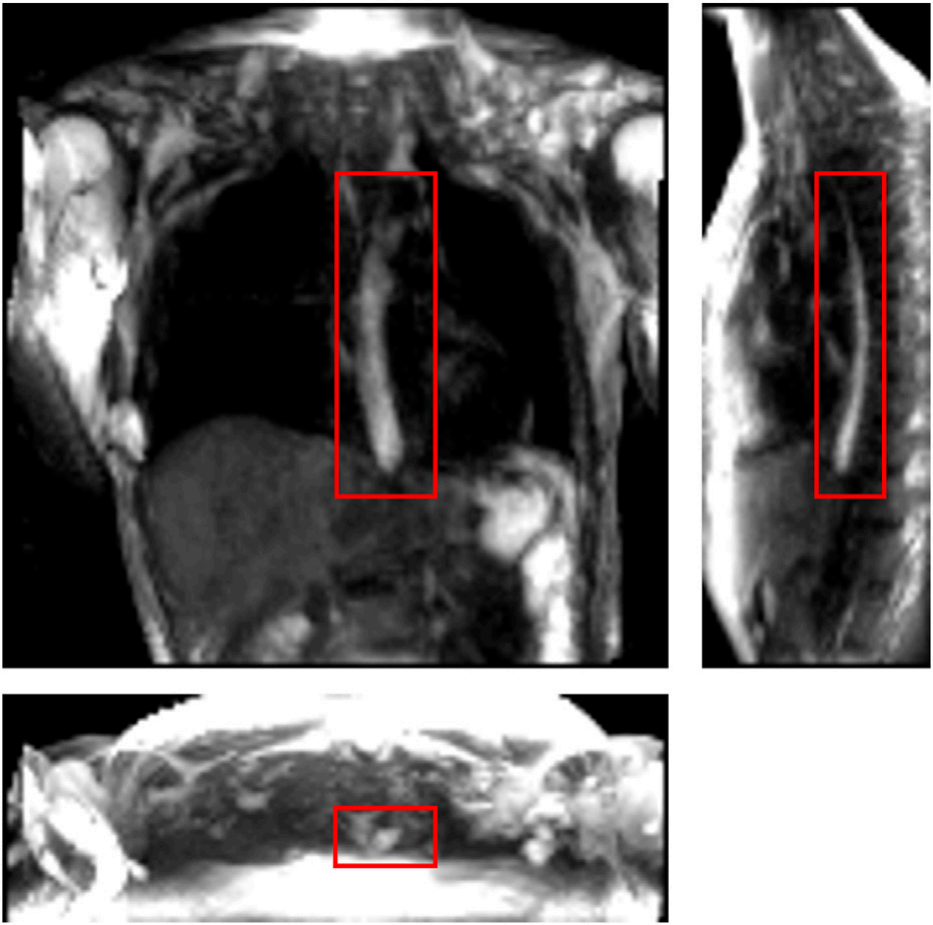
One instance of a dynamic MRI of a normal subject as seen in three perpendicular planes. The planes (from left to right to bottom) are coronal, sagittal, and axial, respectively. The bolus can be seen as the bright region inside the red boxes. Concentrated pineapple juice was swallowed as a contrast agent.

Imaging was performed at 1.5 T (Aera, Siemens, Germany) using a 3D MR angiography sequence (TWIST, Siemens, Germany) designed for contrast-enhanced cardiac imaging applications, which was adapted to be used for esophageal imaging using pineapple juice as an oral contrast agent. Sequence parameters included ${\left(3.25mm\right)}^{3}$ spatial/1.17 s temporal resolution, ${\left(416mm\right)}^{2}\times 143mm$ coronal field of view, 0.78 ms echo time, 2.36 ms repetition time, $29^{o}$ tip angle, 620 Hz/pixel bandwidth, 6/8 partial Fourier acquisition, R = 2 GRAPPA acceleration, and 8% central size/ 10% outer density view sharing. A four-channel cardiac coil was used for image acquisition and placed on the upper torso surface. To improve image conspicuity of the juice bolus, pineapple juice (100 % , Costa Rica) was reduced to a volume factor of 0.48 (i.e., 52 % volume removed) through gradual heating without boiling. By doing so, the T1 of the juice at 1.5 T was reduced from 265 ms (raw/non-volume-reduced juice) to 76 ms (volume-reduced juice), as measured by variable flip angle signal fit. A healthy volunteer was given 20 mL of the volume-reduced pineapple juice to swallow during image acquisition. The juice was administered via a plastic tube and syringe controlled by the scan subject. The subject was instructed to swallow by voice command from the scan operator, given 10 s after the start of image acquisition, with 75 s of imaging performed to capture complete esophageal transit. To visualize the bolus transport, maximum intensity projections were created. Figure 1 shows an instant during bolus transport on three perpendicular slices.

### 2.2 Extraction of bolus geometry

**FIGURE 2:**
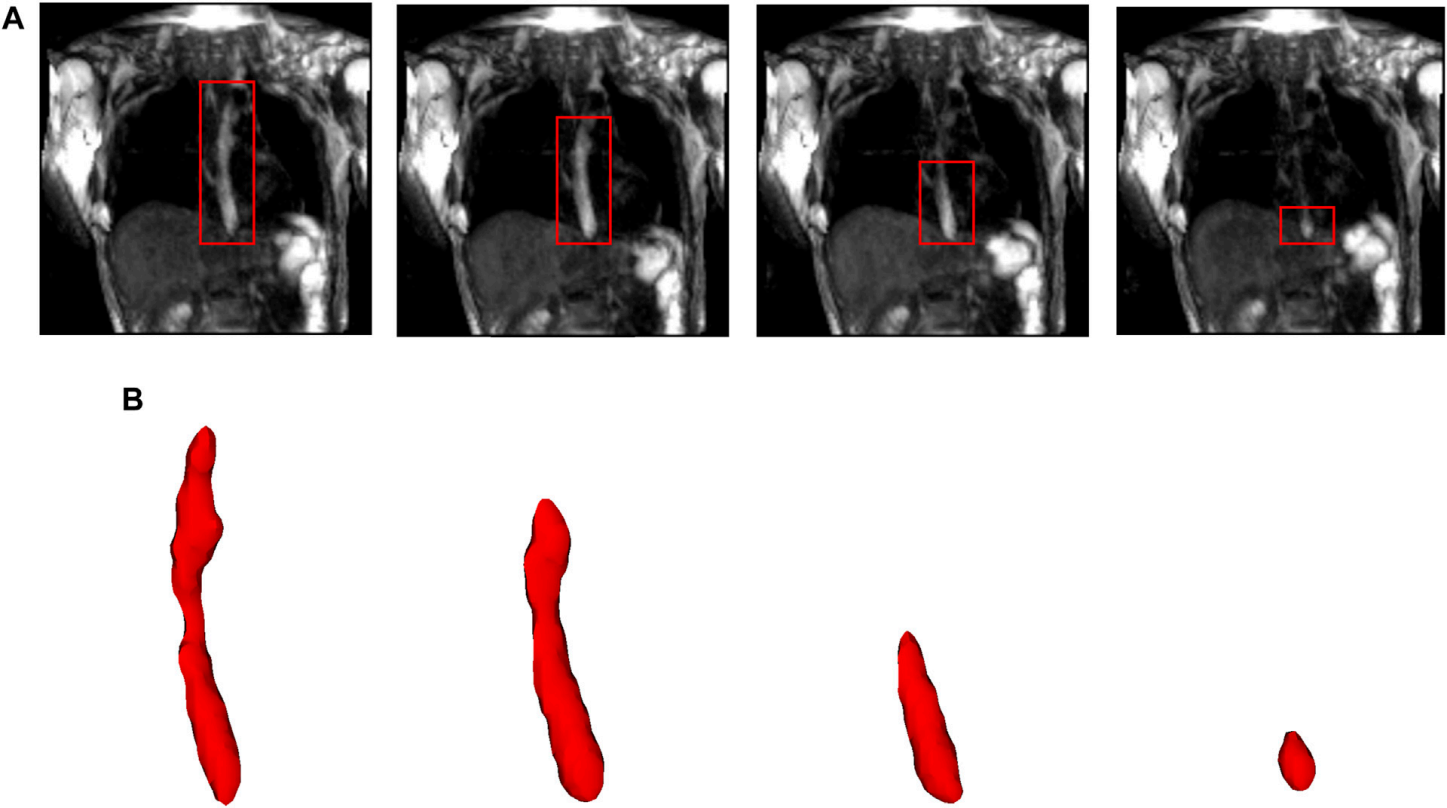
Segmentation of MR images. **(A)** The bolus is shown in the coronal plane at four time instants (progressing from left to right). The bolus is seen as the bright region inside the red boxes. The bolus volume decreased with time as it was emptied into the stomach. **(B)** The corresponding 3D segmented bolus shapes for the four time instants. The bolus size has been magnified for visualization.

In this study, we used data from a single healthy volunteer to demonstrate this framework. The MRI output consisted of a cuboid wherein voxels in a Cartesian coordinate system had different magnitudes of intensity. The temporal resolution of the dynamic MRI (1.17 s) determined the number of images with the bolus seen within the esophagus: seven time instants in this study. The typical length of an adult esophagus is 18–25 cm (Oezcelik and DeMeester, 2011). The average velocity of normal peristalsis is approximately 3.3 cm/s (Hollis and Castell, 1975). Thus, an average swallow sequence usually takes 5–8 s. Therefore, temporal resolutions similar to what we used in our analysis typically result in 5–8 images. Although this temporal resolution is not comparable to fluoroscopy, the detailed three-dimensional geometry of the bolus in MRI leads to better prediction of velocity and intrabolus pressure, resulting in better prediction of esophageal wall properties. The bolus was manually segmented for the seven time instants, a few of which are shown in Figure 2. The segmentation assigned a value of 1 and 0 to each voxel that lay inside and outside the bolus, respectively. The image segmentation was performed using the open-source software ITK-SNAP (Yushkevich et al., 2006). With improved MR imaging and better temporal resolution, manual image segmentation might not be feasible, and more sophisticated automated segmentation techniques might be necessary. We have described in Supplementary Material S1 a deep learning-based automated segmentation approach called 3D-U-Net (Abdulkadir et al., 2016), which was fine-tuned for this application.

**FIGURE 3:**
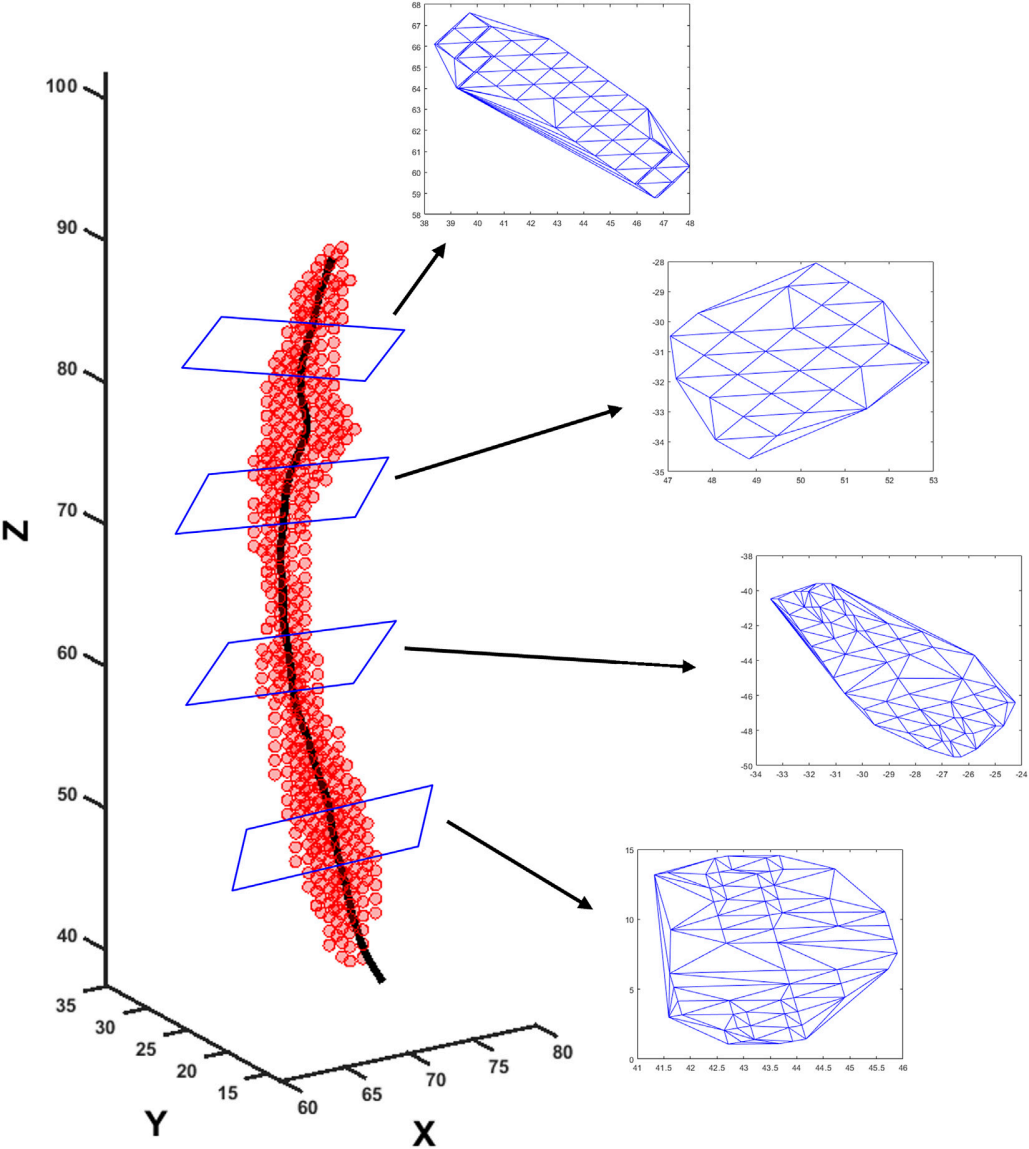
Extraction of cross-sectional areas from dynamic MR images. The segmented bolus geometry at one time instant is shown by the red points in the scatter plot. The generated center line is shown by the black curve inside. A few planes are shown that are perpendicular to the center line and on which the cross-sectional areas were calculated. The points on the planes were meshed using Delaunay triangulation, and the triangulated shapes approximate the cross-sectional areas at those planes.

MRI-MECH modeled the esophagus as a one-dimensional flexible tube. For such a one-dimensional analysis, the variation in cross-sectional areas at different points along the length of the esophagus and at different time instants had to be extracted from the three-dimensional bolus obtained from segmentation. This was performed in two steps. The first step was to generate a center line along the length of the esophagus. The bolus shapes observed at different time instants were superimposed, and then, cross-sections of the superimposed shape at different horizontal planes from the proximal to the distal end of the superimposed shape were generated. The centroids of these cross-sections were connected to form the center line. The length of the center line, in this case, was 9.65 cm. The second step, after extracting the center line, was to generate planes perpendicular to the center line, as shown in Figure 3. The segmented voxels marked 1, which lay near these perpendicular planes, were projected onto these planes. These projected points were connected using Delaunay triangulation, as shown in Figure 3. The cross-sectional area at each point along the center line was then calculated as the sum of the triangles in the Delaunay triangulated geometries.

### 2.3 MRI-MECH formulation

#### 2.3.1 Governing equations

Transport through the esophagus was modeled as a one-dimensional fluid flow through a flexible tube. The mass and momentum conservation equations in one dimension (Barnard et al., 1966; Kamm and Shapiro, 1979; Ottesen, 2003; Manopoulos et al., 2006) are as follows:

$$\begin{array}{c} \frac{\partial A}{\partial t}+\frac{\partial \left(AU\right)}{\partial x}=0, \end{array}$$ (1)

$$\frac{\partial U}{\partial t}+\frac{\partial }{\partial x}\left(\frac{U^{2}}{2}\right)+\frac{1}{\rho }\frac{\partial P}{\partial x}+\frac{8\pi \mu U}{\rho A}=0,$$ (2)

where A is the cross-sectional area of the esophageal lumen and U and P are the velocity and pressure in the bolus fluid, respectively. It should be noted that the fluid pressure P in Eq 2 is relative to the pressure outside the esophagus. Since only pressure gradients are important in Eq 2, the pressure outside the esophagus is not necessary to be known. x represents the distance along the length of the esophagus from the mouth to the stomach, and t represents time. The total time for bolus transport in our analysis was 6.95 s. ρ and μ are the density and dynamic viscosity of the transported fluid, respectively. Pineapple juice was the swallowed fluid, whose density and viscosity were 1.06 g/cm^3^ and 0.003 Pa.s (Shamsudin et al., 2009), respectively.

It has been observed experimentally that the fluid pressure developed inside the esophagus is linearly proportional to the cross-sectional area of the esophageal lumen (Orvar et al., 1993; Kwiatek et al., 2011) in the absence of any neuromuscular activation. Using this information, a pressure tube law can be constructed as follows:

$$P=K\left(\frac{A}{\theta A_{o}}-1\right),$$ (3)

where K is the stiffness of the esophageal wall, $A_{o}$ is the cross-sectional area of the esophageal lumen in its inactive state, and θ is the activation parameter. Typically, the inactive cross-sectional area is in the range of 7–59 ${mm}^{2}$ (Xia et al., 2009). In this case, the inactive cross-sectional area $A_{o}$ was $27\;{mm}^{2}$ , which was manually identified by careful observation of the dynamic MRI. The inactive cross-sectional area may not necessarily be constant along the esophageal length. This is especially true for cases with structural irregularities in the esophageal wall. Since the MRI was performed on a normal subject, there were no major irregularities along the esophageal length, and therefore, assuming a constant inactive cross-sectional area is reasonable. It should be noted that $A_{o}$ might be different for different patients and needs to be determined in a patient-specific manner through dynamic MRIs. The activation parameter θ takes a value of 1 in the inactive state of the esophagus. It can be seen from Eq 3 that in the inactive state when the cross-sectional area of the esophageal lumen is equal to $A_{o}$ , the pressure inside the esophagus relative to the pressure outside the esophagus is equal to 0 mmHg. An activation is induced when θ<1 raises the pressure locally. On the other hand, θ>1 decreases the bolus pressure and estimates the active relaxation of the esophageal wall. Thus, the parameter θ captures the effect of esophageal motility.

Due to the low resolution of the dynamic MRI, it was necessary to interpolate the MRI data to smaller temporal and spatial scales. The measured volume $V_{m}$ of the bolus from the proximal end ( x=0 ) to any point x>0 was calculated as follows:

$$\begin{array}{r} V_{m}=\int_{0}^{x}A_{m}dx \end{array},$$ (4)

where $A_{m}$ is the measured cross-sectional area of the esophageal lumen at a coarse x and t . The volume $V_{m}$ was interpolated using a piecewise cubic Hermite interpolating polynomial to a smaller temporal and spatial scale to obtain V . $V_{o}$ was known at seven time instants and 59 points along x . The interpolated V was calculated at 100 time instants and 100 points along x . Using Eqs 1, 4, the cross-sectional areas and velocities at finer t and x were calculated as follows:

$$A=\frac{\partial V}{\partial x},$$ (5)

$$\begin{array}{c} U=-\frac{1}{A}\frac{\partial V}{\partial t}. \end{array}$$ (6)

The values of A and U were then used to solve for P in Eq. 2. Eqs 1, 2 were non-dimensionalized as follows:

$$\begin{array}{c} \frac{\partial \alpha }{\partial \tau }+\frac{\partial \left(\alpha u\right)}{\partial \chi }=0, \end{array}$$ (7)

$$\begin{array}{c} \frac{\partial u}{\partial \tau }+\frac{\partial }{\partial \chi }\left(\frac{u^{2}}{2}\right)+\frac{\partial p}{\partial \chi }+\varphi \frac{u}{\alpha }=0, \end{array}$$ (8)

where $\alpha =A/A_{s}$ , u=U/c , $\chi =x/\sqrt{A_{s}}$ , $\tau =ct/\sqrt{A_{s}}$ , $p=P/\left(\rho c^{2}\right)$ , $\varphi =\left(8\pi \mu \right)/\left(\rho c\sqrt{A_{s}}\right)$ , $A_{s}=\max\left(A\right)$ , and c=5 cm/s is a reference speed of peristalsis. In this work, $A_{s}=197.73$ ${mm}^{2}$ . Using the properties of the swallowed fluid and the scales for A and U , we found φ=0.101 . The pressure tube law, as described in Eq. 3, was non-dimensionalized as follows:

$$\begin{array}{rr} p & =k\left(\frac{\alpha }{\theta \alpha_{o}}-1\right), \end{array}$$ (9)

where $k=K/\left(\rho c^{2}\right)$ and $\alpha_{o}=A_{o}/A_{s}$ . This non-dimensionalization ensures that the magnitudes of α , u , and p lie between −1 and 1, which is essential for good prediction by the PINN, as described in the following.

#### 2.3.2 Initial and boundary conditions

The boundary conditions of this problem were specified to capture the physiological conditions of normal esophageal transport. The upper esophageal sphincter (UES) at the proximal end of the esophagus opens to allow the bolus into the esophagus, closes once the fluid has passed through it, and remains closed thereafter. Hence, we specified zero velocity at x=0 for all time instants. This condition also ensures that $V_{m}=0$ at x=0 at all time instants and is consistent with Eqs 4, 6. The distal end of the esophagus, on the other hand, remains open to allow emptying into the stomach. Since the pressure term in Eq 2 consists of a single derivative with respect to x , it is necessary to specify only one boundary condition for P . The boundary pressure was specified at the distal end, which is equal to the gastric pressure. It should be noted that the pressure inside the stomach cannot be directly measured through MRI. Thus, it is necessary to use a reference value of 7 mmHg, as reported in the study by De Keulenaer et al. (2009). The stomach pressure may vary from patient to patient as well as at different time instants. However, measuring this pressure would involve the use of catheters with pressure sensors, which in turn would make the procedure invasive. This defeats the purpose of using MRI for diagnosis since it is a safe, non-invasive approach. In practice, the measurement could potentially be made more accurate with the development of standard protocols for diet before MR imaging to ensure consistency, and the assumption of a constant gastric pressure boundary condition will be accurate. Finally, for the initial condition, we assumed zero velocity at all points along x at t=0 .

#### 2.3.3 Cross-sectional area of the lower esophageal sphincter

**FIGURE 4:**
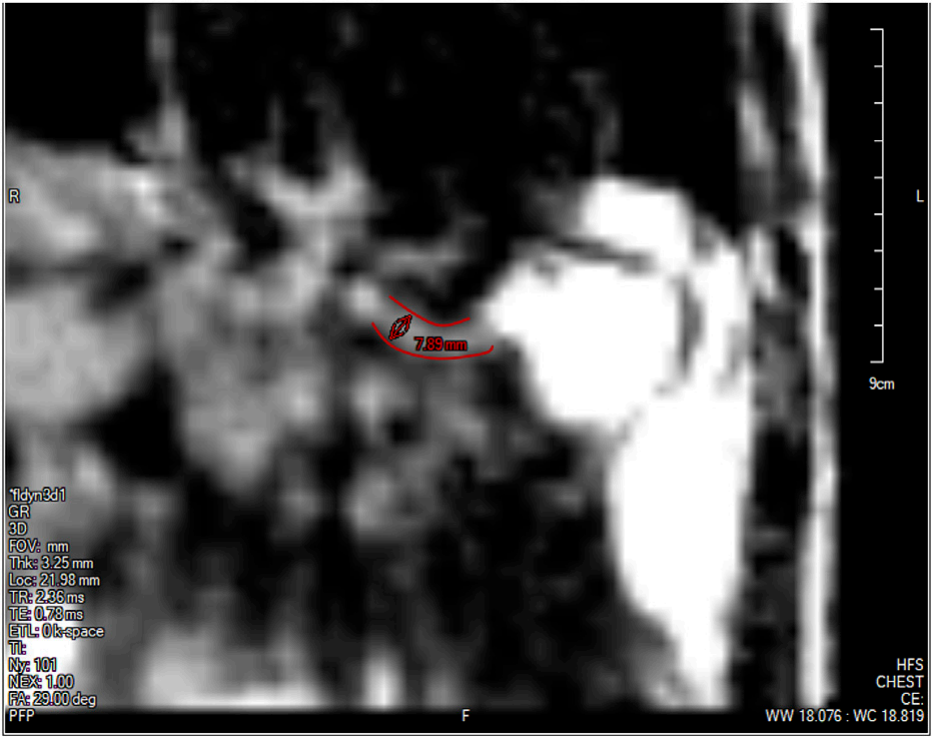
The lower esophageal sphincter identified at a single time instant outlined in red with a diameter of approximately 7.89 mm and a length of approximately 2.78 cm. The stomach can be seen to the right of the LES with the accumulated pineapple juice shown in bright white. The esophageal body cannot be seen in this slice because this plane does not intersect the esophagus.

The low spatial resolution of the dynamic MRI poses a problem in accurately identifying the lower esophageal sphincter (LES) cross-section. This is because the LES opening is narrower than the esophageal body and does not distend very much because of the greater wall stiffness at the EGJ. Although this could be improved by focusing the MRI only on the LES, the state of the esophagus proximal to the LES cannot be estimated in such a scenario. The LES can be identified in only 1 or 2 time instants when the LES has significantly distended due to bolus flow through it. Figure 4 shows the LES at one such time instant. The LES cross-sectional area measured at this time instant can act as a valuable reference to identify the bolus behavior proximal to the LES.

**FIGURE 5:**
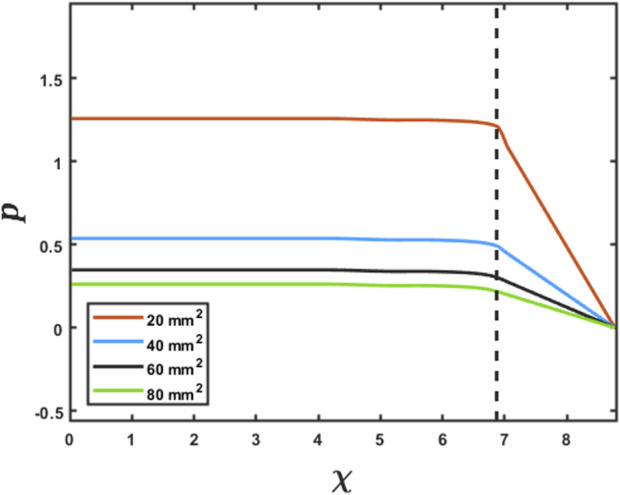
Effect of the LES cross-sectional area on the prediction of intrabolus pressure. The proximal end of the LES is marked by the vertical dashed line. The inserted legend shows LES cross-sectional areas used for this simulation. Eqs 7 and 8 were solved using the method described in FluoroMech (Halder et al., 2021) to calculate the intrabolus pressure. The input for the model was the variation of α observed from the MRI with four reference LES cross-sectional areas. The variation of pressure is shown at a single time instant to illustrate the impact of the LES cross-sectional area on pressure prediction.

As specified in the previous section, since pressure is specified as a Dirichlet boundary condition at the distal end of the esophagus, the intrabolus pressure prediction depends on the accurate measurement of the LES cross-sectional area. Figure 5 shows the intrabolus pressure calculated using the numerical approach described in the study by Halder et al. (2021) with different LES cross-sectional areas. The pressure shown is non-dimensional, and the pressure at the distal end was specified as zero as a reference in this case. The total length of the esophagus considered here is the sum of the center line length (9.65 cm) and the LES length (2.78 cm). Thus, the proximal and distal locations of the bolus were 9.65 cm and 12.43 cm, respectively. In non-dimensional form, the proximal and distal locations were $\chi_{p}=6.87$ and $\chi_{L}=8.81$ , respectively. The quantities $\chi_{p}$ and $\chi_{L}$ were important locations, as described in the next section. As shown in Figure 5, the intrabolus pressure proximal to the LES depends on the LES cross-sectional area, so assuming a constant LES cross-sectional area (measured at one time instant) would lead to an incorrect prediction, making it important to know the instantaneous LES cross-sectional area to accurately predict intrabolus pressure and understand LES functioning during emptying.

#### 2.3.4 Physics-informed neural network

**FIGURE 6:**
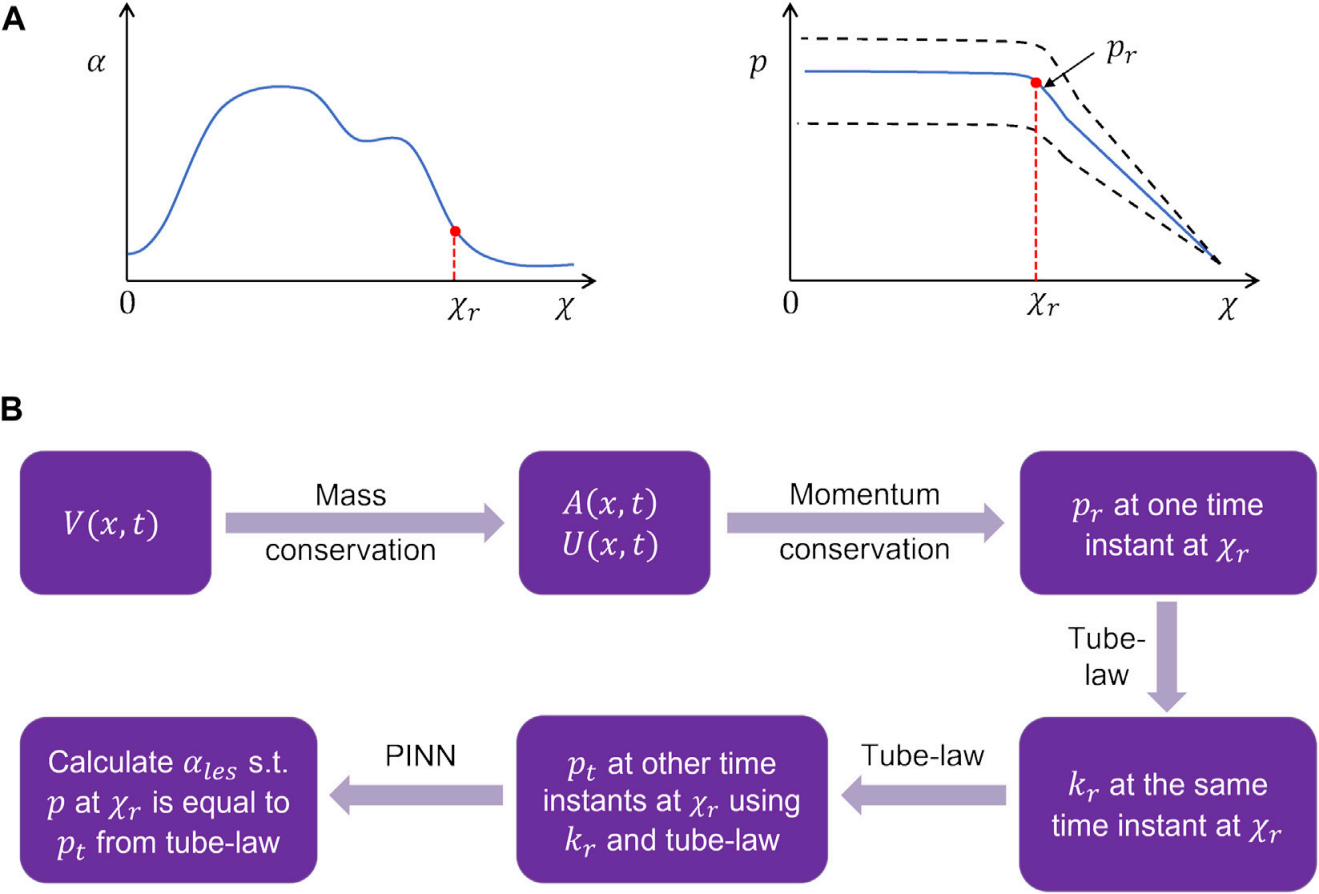
Problem definition for the physics-informed neural network framework. **(A)** Schematic for the variation in the cross-sectional area and pressure at the time instant when the LES cross-section was known. The dashed lines in the pressure variation show what intrabolus pressure would be at other time instants assuming constant LES cross-sectional area. **(B)** Workflow for the prediction of the LES cross-sectional area at other time instants.

The problem of missing data for the LES cross-sectional area (and consequently obtaining accurate intrabolus pressure values) was solved using a physics-informed neural network (Raissi et al., 2019). The problem description is schematically shown in Figure 6. The final interpolated volume $V\left(x,t\right)$ was used to calculate $A\left(x,t\right)$ and $U\left(x,t\right)$ using Eqs 5, 6 and after non-dimensionalization, $\alpha \left(\chi ,\tau \right)$ and $u\left(\chi ,\tau \right)$ , respectively. These values of $\alpha \left(\chi ,\tau \right)$ and $u\left(\chi ,\tau \right)$ were then used to calculate $p\left(\chi_{r},\tau_{r}\right)$ at the specific time instant when the LES cross-section was visible by solving Eq 8 using the finite volume method described in the study by Halder et al. (2021). The non-dimensional time $\tau_{r}$ corresponds to the time instant when the LES was visible in MRI. The point $\chi_{r}$ was selected near the proximal end of the LES. This point was selected because the pressure at points proximal to $\chi_{r}$ is of similar magnitude as $p\left(\chi_{r},\tau \right)$ , as shown in Figure 5. Additionally, $\chi_{r}$ was very close to the LES, and hence, the effect of active relaxation as observed in the esophageal body was minimal. It should be noted that this was an assumption that we made regarding active relaxation, and its usefulness will be explained shortly. The values of $\chi_{r}$ and $\tau_{r}$ were 6.76 and 8.57, respectively. The pressure $p\left(\chi_{r},\tau_{r}\right)$ was the correct estimate of the intrabolus pressure since the LES cross-sectional area was accurately known. We call this pressure the reference pressure, $p_{r}=p\left(\chi_{r},\tau_{r}\right)$ . Using the tube law in Eq 9, the stiffness ( $k_{r}$ ) at $\chi_{r}$ was calculated as follows:

$$\begin{array}{r} k_{r}=\frac{p_{r}}{\left(\frac{\alpha \left(\chi_{r},\tau_{r}\right)}{\alpha_{o}}-1\right)}. \end{array}$$ (10)

It should be noted that there is no θ in Eq 10 since we assumed that θ=1 at $\chi_{r}$ . With the stiffness at $\chi_{r}$ known, we calculated the pressure $p_{t}=p\left(\chi_{r},\tau \right)$ at other times with the tube law according to Eq 9 as follows:

$$\begin{array}{r} p_{t}=k_{r}\left(\frac{\alpha \left(\chi_{r},\tau \right)}{\alpha_{o}}-1\right). \end{array}$$ (11)

The LES cross-sectional area ( $A_{les}$ ) was calculated using the PINN so that the pressure predicted at $\chi_{r}$ matches $p_{t}$ for all times. An additional constraint is necessary to ensure a unique solution for $A_{les}$ as follows:

$$\begin{array}{r} \frac{\partial \alpha_{les}}{\partial \chi }=0 \end{array},$$ (12)

where $\alpha_{les}=A_{les}/A_{s}$ is the non-dimensional cross-sectional area of the LES. Equation 12 implies that there was no significant variation in the LES cross-sectional area along χ . This is physically meaningful since the variation of $\alpha_{les}$ along χ is quite negligible compared to the esophageal body and can also be observed in Figure 4. It should be noted that assuming $\partial \alpha_{les}/\partial \chi =0$ can lead to discontinuity at $\chi =\chi_{p}$ . The PINN uses tanh activation functions at every hidden unit in each of the hidden layers, which transforms χ and τ to predict α , u , and p . This leads to smoothening at potentially discontinuous locations in χ and τ and, thus, enables the predictions ( α , u , and p ) to be differentiable. However, since there are multiple layers in the PINN, the multiple nonlinear transformations through tanh activations also enable the PINN to fit nearly discontinuous solutions very closely, as described in the study by Raissi et al. (2019). Thus, any artificial smoothening is minimized.

##### 2.3.4.1 Network architecture

**FIGURE 7:**
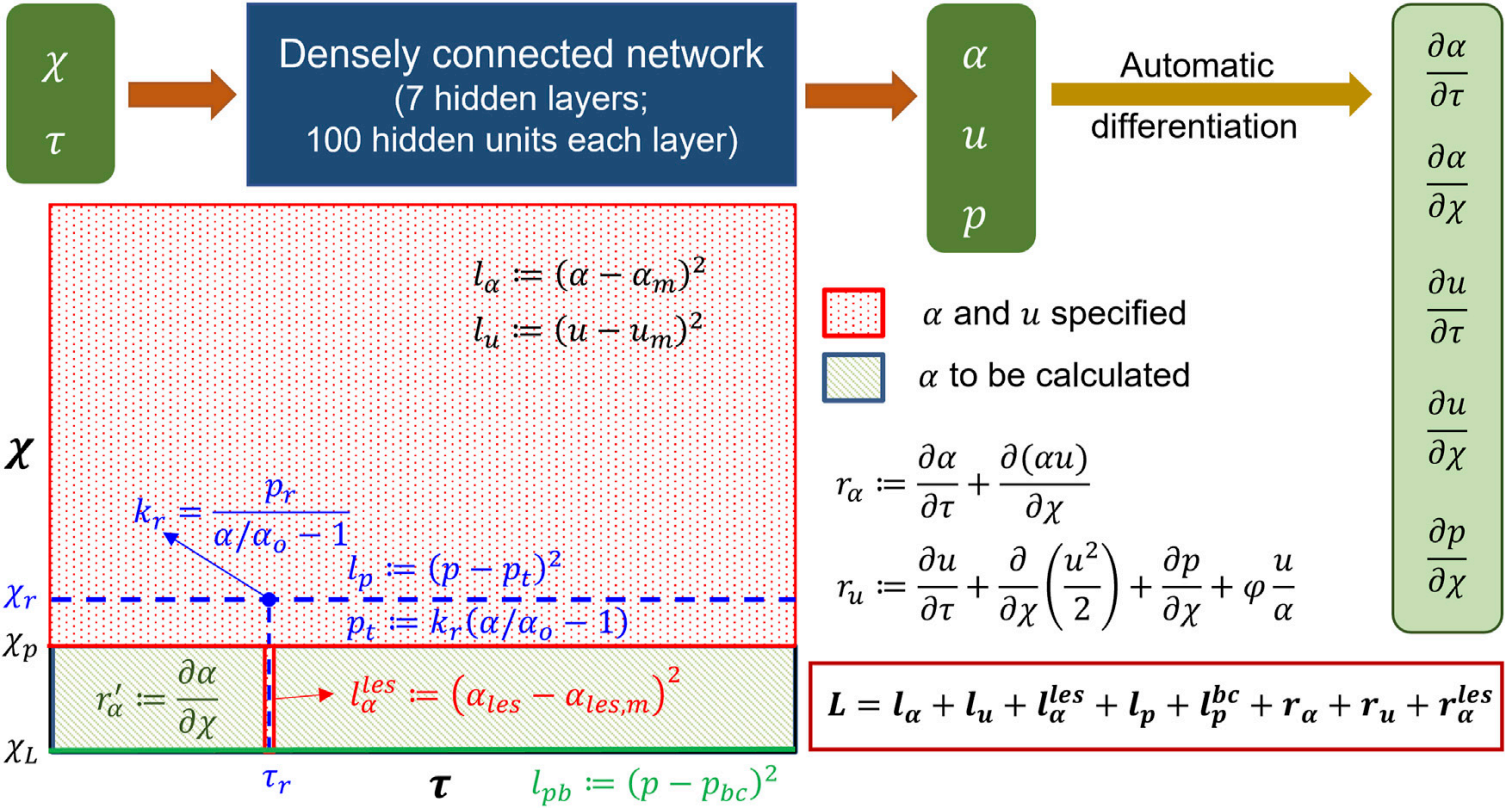
Details of the physics-informed neural network. The input and output of the PINN along with the details of the hidden layers are shown at the top. Automatic differentiation was used to calculate the derivative terms for the residuals. The schematic of the domain is shown in the following. The schematic describes where the different losses were specified.

The schematic in Figure 7 shows the architecture of the PINN. It takes χ and τ as input and predicts α , u , and p . Since the inputs are χ and τ , automatic differentiation can be effectively used to calculate $\frac{\partial \alpha }{\partial \tau }$ , $\frac{\partial \alpha }{\partial \chi }$ , $\frac{\partial u}{\partial \tau }$ , $\frac{\partial u}{\partial \chi }$ , and $\frac{\partial p}{\partial \chi }$ , which were used for calculating the terms in Eqs 7, 8. In addition to the input and output layers, the PINN consisted of seven hidden layers with 100 hidden units in each layer. We used the tanh activation function for every layer.

##### 2.3.4.2 Losses

The losses for the PINN consisted of a combination of measurement losses and residuals of the mass and momentum conservation equations. Minimizing the measurement losses ensures that the solutions are consistent with the measurements, and minimizing the residuals ensures that the governing physics behind the problem is followed. Figure 7 shows the locations and time instants at which the different measurement losses and residuals were calculated. As already mentioned in the workflow, α and u were known at all points proximal to the bolus (marked in red) for all time instants. The measurement losses for α and u for $\chi <\chi_{p}$ and $0\leq \tau \leq \tau_{T}$ were as follows:

$$\begin{array}{c} l_{\alpha }=\frac{1}{N_{1}}\sum_{i=1}^{N_{1}}{\left(\alpha^{i}-\alpha_{m}^{i}\right)}^{2}, \end{array}$$ (13)

$$\begin{array}{c} l_{u}=\frac{1}{N_{1}}\sum_{i=1}^{N_{1}}{\left(u^{i}-u_{m}^{i}\right)}^{2} \end{array},$$ (14)

where the quantities with the subscript m represent measured quantities. $\chi_{p}$ is the proximal end of the LES, and $\tau_{T}$ is the total time (non-dimensional) of bolus transport. Each point i was taken from a Cartesian grid of 99 nodes along τ and 100 nodes along χ , which leads to $N_{1}=9900$ . It should be noted that we are calling $u_{m}$ as a measured quantity for the PINN, although we calculate it along with α through the interpolated volume V , as described in Section 2.3.1. This is because the PINN minimizes the square of the difference between the prediction of α and u from the network and their already known values (which are analogous to measurements because they are already known quantities for the PINN). Additionally, the LES cross-sectional area was known at $\tau =\tau_{r}$ for $\chi_{p}<\chi \leq \chi_{L}$ , and the corresponding measurement loss was as follows:

$$\begin{array}{rr} l_{\alpha }^{les} & =\frac{1}{N_{2}}\sum_{i=1}^{N_{2}}{\left(\alpha_{les}^{i}-\alpha_{les,m}^{i}\right)}^{2} \end{array},$$ (15)

where $\chi_{L}$ is the non-dimensional coordinate of the distal end. The points i were taken from a uniform mesh of $N_{2}=28$ points along χ at $\tau_{r}$ . The measurement loss for pressure was calculated at $\chi =\chi_{r}$ for τ≥0 and was defined as follows:

$$\begin{array}{r} l_{p}=\frac{1}{N_{3}}\sum_{i=1}^{N_{3}}{\left(p^{i}-p_{t}^{i}\right)}^{2} \end{array},$$ (16)

where the point i was selected from a uniform mesh of $N_{3}=98$ along τ at $\chi =\chi_{r}$ . Additionally, the Dirichlet pressure boundary condition was enforced at $\chi =\chi_{L}$ for τ≥0 through the following loss:

$$\begin{array}{r} l_{p}^{bc}=\frac{1}{N_{4}}\sum_{i=1}^{N_{4}}{\left(p^{i}-p_{bc}^{i}\right)}^{2}, \end{array}$$ (17)

where $p_{bc}$ is the pressure specified at the distal end of the esophagus and $N_{4}=99$ with i selected from a uniform grid along τ . The residual losses were calculated in the entire domain for $0\leq \chi \leq \chi_{L}$ and τ≥0 according to Eqs 7, 8 as follows:

$$\begin{array}{c} r_{\alpha }=\frac{1}{N_{5}}\sum_{i=1}^{N_{5}}\left[\frac{\partial \alpha^{i}}{\partial \tau }+\frac{\partial \left(\alpha^{i}u^{i}\right)}{\partial \chi }\right], \end{array}$$ (18)

$$\begin{array}{c} r_{u}=\frac{1}{N_{5}}\sum_{i=1}^{N_{5}}\left[\frac{\partial u^{i}}{\partial \tau }+\frac{\partial }{\partial \chi }\left(\frac{{\left(u^{i}\right)}^{2}}{2}\right)+\frac{\partial p^{i}}{\partial \chi }+\varphi \frac{u^{i}}{\alpha^{i}}\right], \end{array}$$ (19)

where i was randomly sampled from a uniform distribution of points in the entire domain with $N_{5}=50688$ . Finally, the constraint, as described in Equation 12, led to the following residual:

$$\begin{array}{r} r_{\alpha }^{les}=\frac{1}{N_{6}}\sum_{i=1}^{N_{6}}\frac{\partial \alpha_{les}^{i}}{\partial \chi }=0 \end{array},$$ (20)

where i was randomly sampled from a uniform distribution of points in the domain $\left[\chi_{p},\chi_{L}\right]$ and $\left[0,\tau_{T}\right]$ with $N_{6}=5544$ . The total loss for the PINN was the sum of all the measurement losses and residuals as follows:

$$\begin{array}{r} L=l_{\alpha }+l_{u}+l_{\alpha }^{les}+l_{p}+l_{p}^{bc}+r_{\alpha }+r_{u}+r_{\alpha }^{les}. \end{array}$$ (21)

To train the network, the inputs χ and τ were normalized with their mean and standard deviation as follows:

$$\begin{array}{c} \chi^{\prime }=\frac{\chi -\mu_{\chi }}{\sigma_{\chi }} \end{array},$$ (22)

$$\begin{array}{c} \tau^{\prime }=\frac{\tau -\mu_{\tau }}{\sigma_{\tau }}, \end{array}$$ (23)

where μ and σ are the corresponding mean and standard deviations, respectively, for χ and τ . Hence, the derivatives with respect to χ and τ gets modified as follows:

$$\frac{\partial }{\partial \chi }\left(\cdot \right)=\frac{1}{\sigma_{\chi }}\frac{\partial }{\partial \chi^{\prime }}\left(\cdot \right),$$ (24)

$$\begin{array}{c} \frac{\partial }{\partial \tau }\left(\cdot \right)=\frac{1}{\sigma_{\tau }}\frac{\partial }{\partial \tau^{\prime }}\left(\cdot \right) \end{array}.$$ (25)

##### 2.3.4.3 Training

**FIGURE 8:**
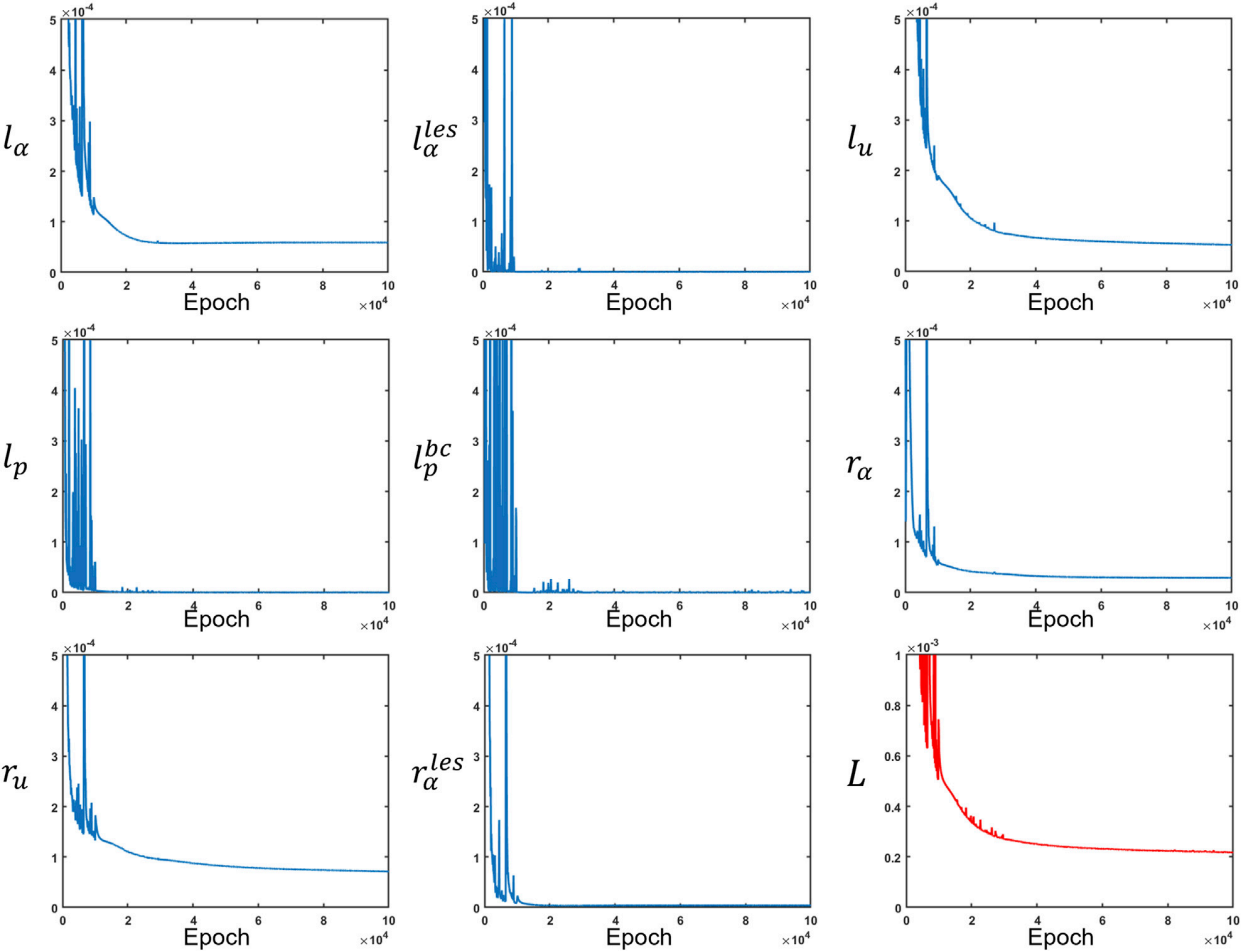
Measurement losses and residuals along with the total loss. All loss functions were minimized at different rates. The total loss is depicted in red, while the other losses are in blue.

The network was trained using TensorFlow (MaA et al., 2016) for 100000 epochs. We used an Adam (DPaB, 2014) optimizer to minimize the losses. A piecewise constant decayed learning rate was used to minimize the losses efficiently. The learning rate was 0.001 for the first 10000 epochs, 0.0001 for the next 20000 epochs, and 0.00003 for the last 70000 epochs. The final values for $l_{\alpha }$ , $l_{u}$ , $l_{\alpha }^{les}$ , $l_{p}$ , $l_{p}^{bc}$ , $r_{\alpha }$ , $r_{u}$ , and $r_{\alpha }^{les}$ were $5.9\times 10^{-5}$ , $9.8\times 10^{-7}$ , $5.3\times 10^{-5}$ , $4.0\times 10^{-7}$ , $2.7\times 10^{-7}$ , $2.9\times 10^{-5}$ , $7.2\times 10^{-5}$ , and $3.8\times 10^{-6}$ , respectively. Figure 8 shows the learning curves for the various loss functions. The final total loss was $2.2\times 10^{-4}$ .

##### 2.3.4.4 Verification using the method of manufactured solutions

The PINN predictions of α , u , and p , given a set of χ and τ as input, were verified using the method of manufactured solutions (MMS) for the same set of governing equations, i.e., Eqs 7–9. The assumed cross-sectional area for verification was as follows:

$$\begin{array}{c} f\left(\chi \right)=\frac{256}{9}\left[2\chi^{5}-5\chi^{4}+4\chi^{3}-\chi^{2}\right]+1, \end{array}$$ (26)

$$\begin{array}{c} \alpha =\frac{1}{2}\left[\left(f-1\right)\cos\left(w\tau \right)+1\right], \end{array}$$ (27)

where w = 5, $\chi \in \left[0,1\right]$ , and $\tau \in \left[0,1\right]$ . Solving Eq 7 provides the following analytic expression for u :

$$\begin{array}{c} u=\frac{128w\sin\left(wt\right)}{9\alpha }\left[\frac{\chi^{6}}{3}-\chi^{5}+\chi^{4}-\frac{\chi^{3}}{3}\right]. \end{array}$$ (28)

**FIGURE 9:**
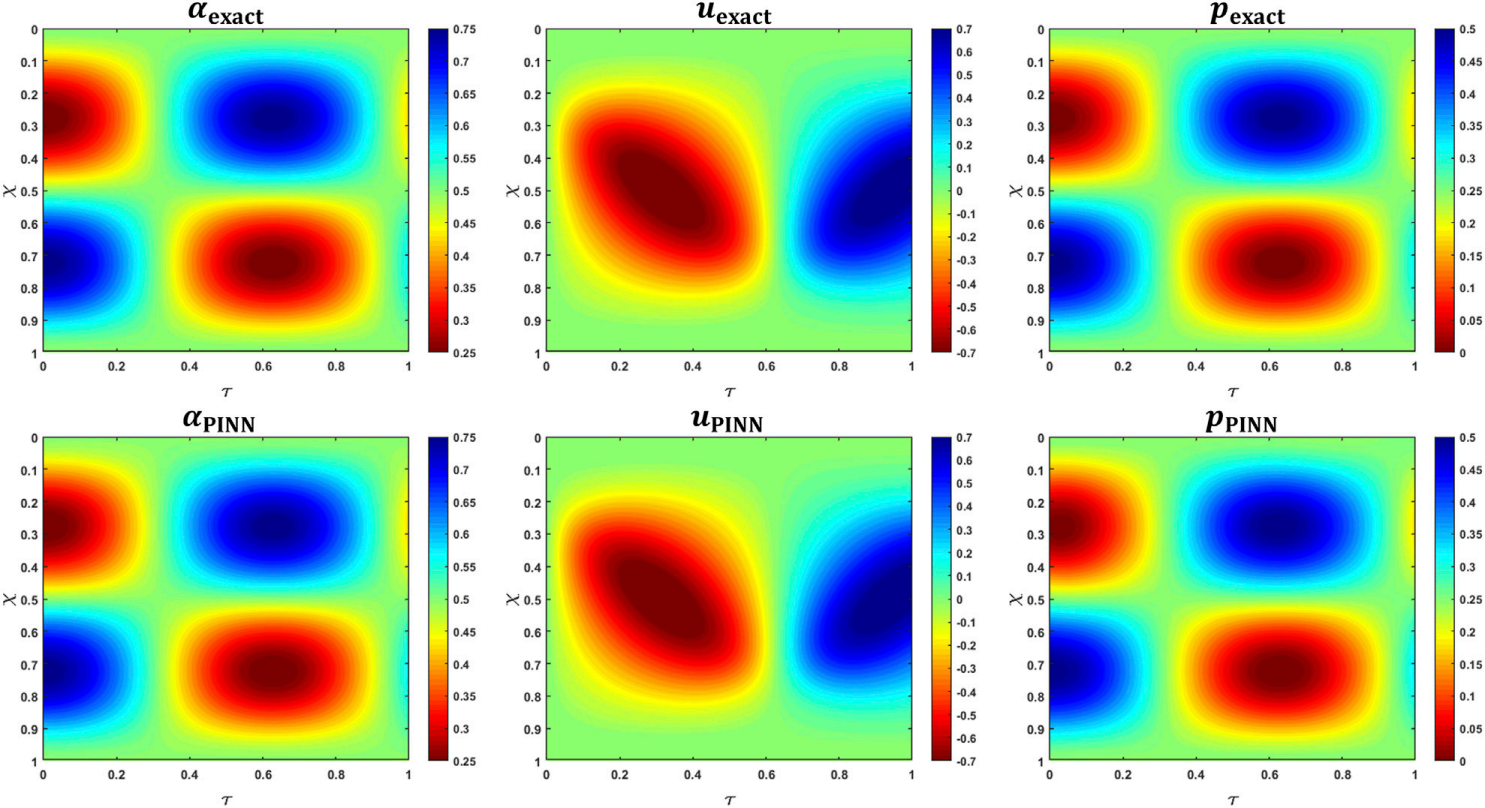
MMS verification of PINN predictions. The top row shows the variations in the analytical expressions for α , u , and p . The bottom row shows the corresponding variations as predicted by the PINN. The $L_{2}$ error for α , u , and p was $3.2\times 10^{-4}$ , $2.2\times 10^{-4}$ , and $1.6\times 10^{-3}$ , respectively.

Finally, we assumed an expression for pressure according to Eq 9 with θ=1,k=0.25, and $\alpha_{o}=0.25$ . With these expressions for α,u, and p and assuming φ=0.1 , we get a source term on the right-hand side of Eq 8. The total loss to be minimized for this problem is the sum of the measurement losses and residues described by Eqs 13, 14, 17–19. The measurement losses were calculated on a grid of 99 points along τ and 100 points along χ exactly like the description in Section 2.3.4.2, leading to $N_{1}=9900$ , as described in Eqs 13, 14. For the residues, points in the computational domain were generated following the same approach, as described in Section 2.3.4.2, resulting in $N_{5}=50688$ . The network architecture and training parameters were kept the same, as described in Figure 7 and Section 2.3.4.3, respectively. The final values for $l_{\alpha }$ , $l_{u}$ , $l_{p}^{bc}$ , $r_{\alpha }$ , and $r_{u}$ were $1.7\times 10^{-7}$ , $1.5\times 10^{-7}$ , $1.4\times 10^{-7}$ , $3.9\times 10^{-7}$ , and $6.7\times 10^{-7}$ , respectively. As shown in Figure 9, the predicted solutions closely match the exact solutions, with the $L_{2}$ error for α , u , and p being $3.2\times 10^{-4}$ , $2.2\times 10^{-4}$ , and $1.6\times 10^{-3}$ , respectively.

#### 2.3.5 Esophageal wall stiffness and active relaxation

The esophageal wall stiffness and active relaxation were calculated as described in the study by Halder et al. (2021). A few manipulations of Eq 3 yield the following:

$$\begin{array}{r} \frac{P}{\frac{A}{A_{o}}-1}=\frac{K}{\theta }\left[1-\frac{\theta -1}{\frac{A}{A_{o}}-1}\right]. \end{array}$$ (29)

The active relaxation parameter θ is always greater than 1 at the location of the bolus. Additionally, $A>A_{o}$ at the bolus due to the distension of esophageal walls. Thus, the second term of Eq. 29 is always greater than 0. Using these constraints, we arrive at the following inequality:

$$\begin{array}{r} \frac{K}{\theta }\geq \frac{P}{\frac{A}{A_{o}}-1}, \end{array}$$ (30)

where K/θ estimates the lower bound of the esophageal stiffness and also incorporates the effect of active relaxation. The active relaxation of the esophageal walls was estimated as follows:

$$\begin{array}{r} \theta =\frac{\alpha }{\alpha_{r}}, \end{array}$$ (31)

where $\alpha_{r}$ is the reference non-dimensional cross-sectional area near the distal end of the esophageal body at $\chi =\chi_{r}$ , as shown in Figure 6. The value of θ at $\chi_{r}$ was assumed to be 1 and acted as a reference to calculate active relaxation for all $\chi <\chi_{r}$ .

## 3 Results and discussion

**FIGURE 10:**
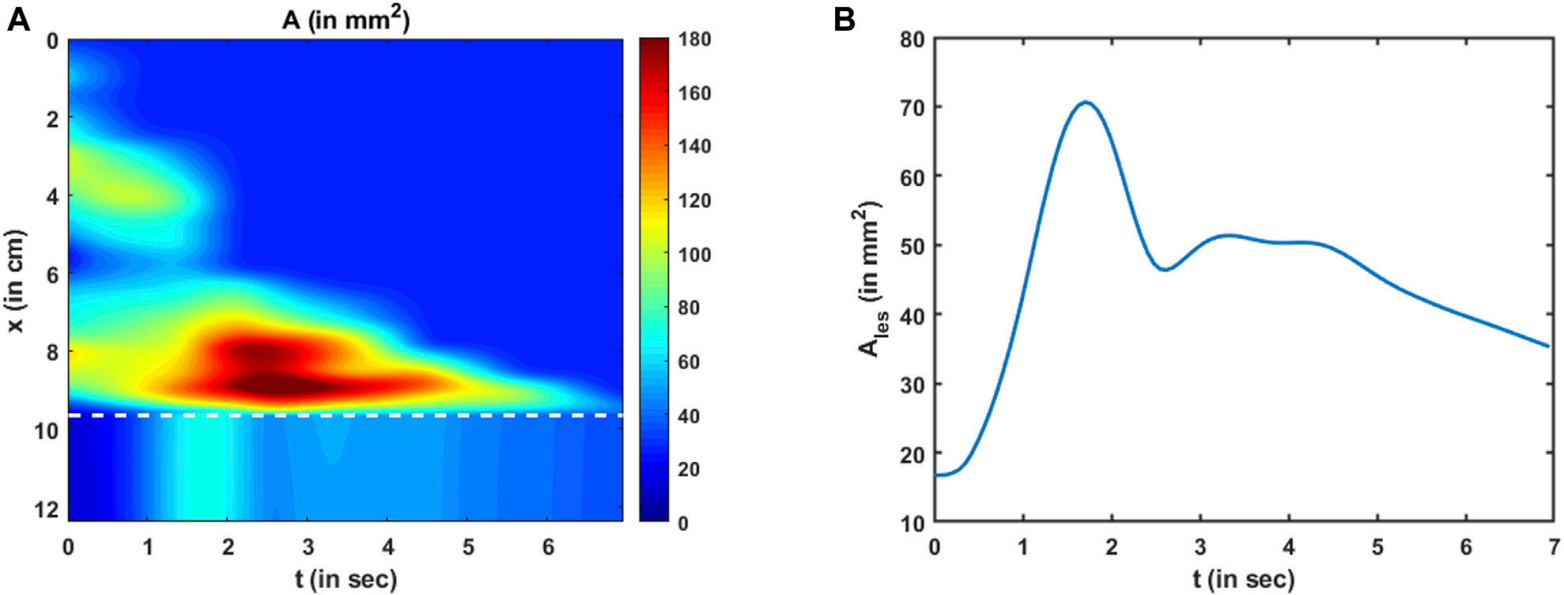
Variation in the cross-sectional area as predicted by the PINN. **(A)** Variation in A as a function of x and t. The dashed white line indicates the proximal end of the LES. The cross-sectional area above the dashed line was known from MRI, and its prediction by the PINN was ensured by minimizing Eq 13. There is no variation in A along x within the LES due to the constraint described in Eq 12, **(B)** Variation in the LES cross-sectional area as a function of time. It had the greatest magnitude near the instant such that the LES was visible in the MRI image.

The PINN predicts the non-dimensional cross-sectional area, fluid velocity, and fluid pressure by minimizing a set of measurement losses and ensuring that the physics of the fluid flow problem is followed throughout. The variation in the predicted cross-sectional area (in its dimensional form) as a function of x and t is shown in Figure 10A. The values of the cross-sectional areas inside the bolus proximal to the LES were obtained from measurements, and their prediction was based on the minimization of the measurement loss, as described in Eq 13.

**FIGURE 11:**
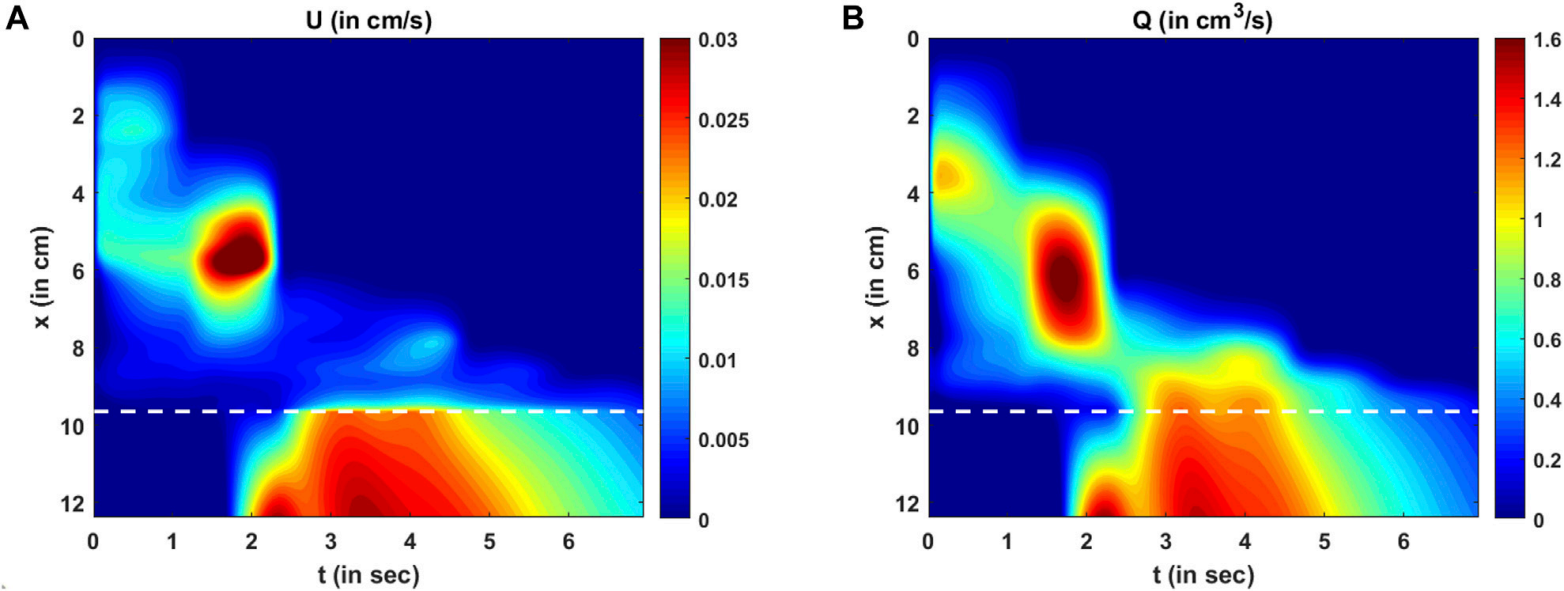
Variations in velocity and flow rate. **(A)** Variation in U as predicted by the PINN. There are two high-velocity zones: one at x = 6 cm, t = 2 s and the other at the LES for t > 2 s. These high-velocity zones match the regions of low cross-sectional areas. **(B)** Variation in the mean flow rate calculated as Q = AU. The high flow rate matches the high-velocity zones, but there is a smoother transition of Q at the proximal end of the LES compared to U.

The cross-sectional areas proximal to the bolus cannot be visualized in MRI because the fluid contrast medium was completely displaced by the peristaltic contraction, and dynamic MR imaging cannot distinguish the esophagus from surrounding tissues. Hence, we assigned the inactive cross-sectional area $A_{o}$ to the esophagus proximal to the bolus. We found that this assignment does not impact the prediction of any of the physical quantities using the PINN. This is because the velocity (and flow rate) proximal to the bolus is automatically predicted as zero (as shown in Figures 11A, B) with this assignment, and since the pressure boundary condition is specified at the distal end, the pressure calculation inside the domain does not depend on the behavior proximal to the bolus. The variation in the LES cross-sectional area can be seen below the dashed line in Figure 10A. The LES cross-sectional area does not vary along x and only varies along t . This is because we enforced the constraint, as described in Eq 12.

The variation in the LES cross-sectional area is shown more clearly in Figure 10B. The prediction of $A_{les}$ depends on the reference LES cross-sectional area observed at a single time instant, the conservation laws, and the reference pressure prediction at $\chi_{r}$ . $A_{les}$ has the greatest magnitude near the instant when the LES cross-sectional area was observed in the MRI image and has lesser values farther away from that instant. This matches our observation from the MRI images that the LES could not be visualized most of the time. Hence, since the effectiveness of esophageal transport essentially depends on how effectively the esophagus empties, the LES cross-sectional area is an important physiomarker of esophageal function. A greater LES cross-sectional area facilitates esophageal emptying, while it becomes unnecessary for the LES to have a large cross-sectional area when the bolus has almost completely emptied. Similar LES behavior is evident in Figure 10B, where it was greater during the emptying process and minimal when bolus emptying was nearly complete.

The variations in bolus fluid velocity and flow rate are shown in Figures 11A, B, respectively. There are two major high-velocity zones. The first high-velocity zone is near x=6 cm at t=2 sec. Comparing this region with Figure 10A, it is evident that the cross-sectional area at that location and time was less than at its adjacent regions. The second high-velocity zone was in the LES. This also corresponds to a low cross-sectional area. Thus, the velocities are greater at lower cross-sectional areas, which is intuitive for low-viscosity fluids. The flow rate is the rate at which the bolus is emptied out of the esophagus, and zones with high flow rates are similar to those with high velocity. However, there is a smoother transition of the flow rate from the esophageal body to the LES than to the velocity field. This is because the LES cross-sectional area was much smaller than that of the esophageal body, requiring the fluid velocity to increase more to maintain the same flow rate.

**FIGURE 12:**
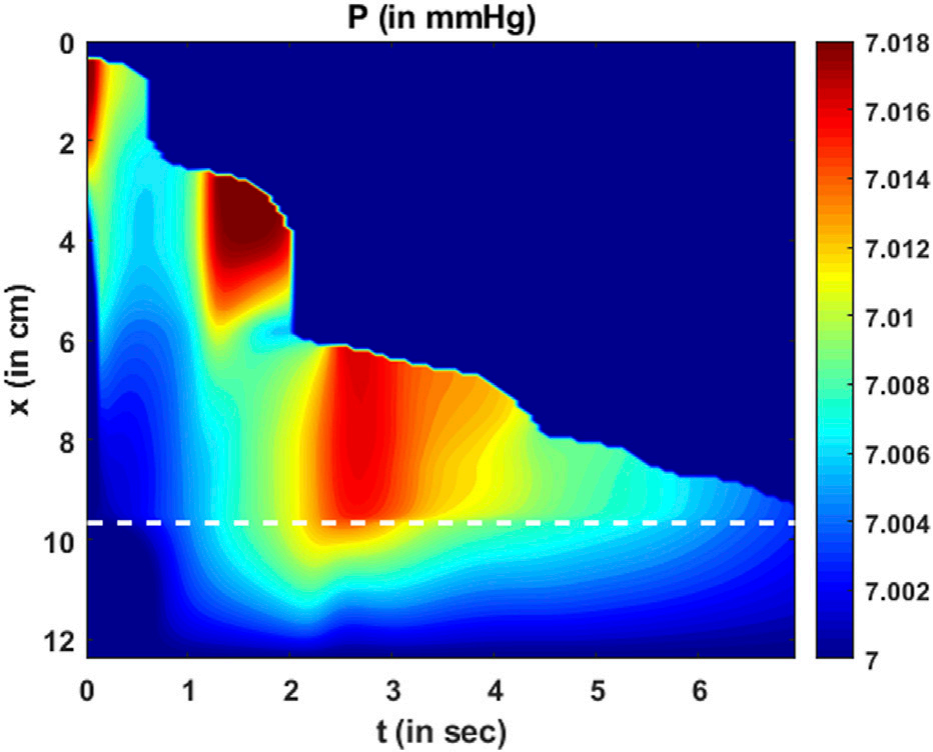
Variation in pressure as a function of x and t . Two major high-pressure zones can be identified wherein the fluid locally accelerates making the corresponding fluid velocity greater in those regions. It should be noted that the magnitude of dynamic pressure variations is minimal compared to the total pressure.

The variation in fluid pressure is shown in Figure 12. The pressure gradients along x drive the fluid through the esophagus. Comparing Figures 11A; Figure 12, we can observe that the high-pressure gradients match the high-velocity zones. This is because the high-pressure gradients locally accelerate the fluid. It should be noted that the pressure variations are minimal compared to the magnitude of the pressure. An intragastric pressure of 7 mmHg was used as a boundary condition for pressure at the distal end, which is in the normal range for a healthy subject. The thoracic pressure was assumed to be 0 mmHg. Thus, the intrabolus pressure must be greater than the intragastric pressure to empty into the stomach. The major portion of this pressure ( ∼ 7 mmHg) is developed by the elastic distention of the esophageal walls. A small portion of the total intrabolus pressure ( ∼ 0.01 mmHg) is attributable to the local acceleration or deceleration of the bolus fluid. Since MRI shows only the movement within an already distended esophagus, the calculated pressure variations are minimal and correspond to local acceleration or deceleration of the fluid. This observation regarding dynamic pressure variations was also observed in a mechanics-based analysis of fluoroscopy (Halder et al., 2021).

The total intrabolus pressure, as shown in Figure 12, is within the normal range according to CCv4.0, leading us to conclude that our specifications for intragastric pressure and thoracic pressure were valid. The prediction of $A_{les}$ depends on the pressure gradients and not on the actual magnitude of the pressure. Therefore, the prediction of $A_{les}$ remains the same, irrespective of the boundary condition chosen for P . Figure 10B; Figure 11A, B; Figure 12 also point at an important feature of the LES. The greatest LES cross-sectional area (at approximately 1.8 s) neither matches the greatest pressure nor the greatest velocity (or flow rate) across the LES. This demonstrates that the LES opening is not governed passively by intrabolus pressure. If the LES was passively opened by elastic distention due to the intrabolus pressure, then the maximum LES cross-sectional area would coincide with the maximum pressure gradient. Since that is not observed, it can be concluded that the LES cross-sectional area also involves neuromuscular relaxation.

**FIGURE 13:**
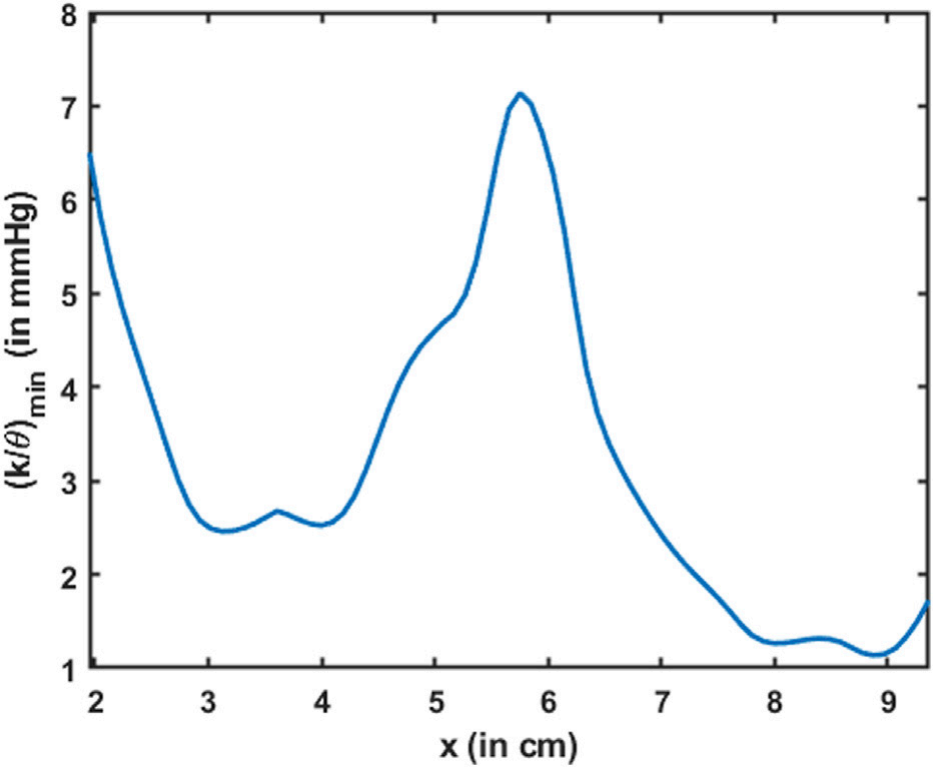
Variation in the minimum esophageal wall stiffness along the length of the esophagus. This measure of stiffness accounts for active relaxation and captures the wall characteristics when the esophagus was distended. The stiffness is shown only for the esophageal body proximal to the LES.

Esophageal wall stiffness (along with the effect of active relaxation) was estimated by the parameter K/θ . The minimum value of K/θ corresponds to the lower bound of the effective stiffness of the esophageal walls when distended. Since the cross-sectional area of the esophagus is not visible in MRI, any prediction regarding the stiffness at those locations would be inaccurate. Hence, predictions of wall stiffness can only be made at regions where the esophagus is distended, i.e., at the location of the bolus. However, the distended esophageal walls also undergo active relaxation to accommodate an incoming bolus and minimize intrabolus pressure. The combined behavior of passive elastic distention of the esophageal walls and active relaxation is captured by the parameter K/θ . Since K/θ , as described by Eq 30, estimates the lower bound of the effective esophageal stiffness, the most accurate estimate of K/θ occurs when the esophageal walls are most distended. The maximum distension corresponds to the minimum value of K/θ , which is shown in Figure 13. The minimum K/θ at each x was calculated for all values of t . It should be noted that the high value of ${\left(K/\theta \right)}_{\min}$ near x=6 cm in Figure 13 matches the low cross-sectional area region in Figure 10A. This makes sense because the esophagus would distend less at locations of greater stiffness. It should be noted that although the stiffness appears high at x=6 cm, it does not necessarily mean that the esophageal tissue is stiffer at that location. When the esophageal wall comes in contact with surrounding organs, it appears stiffer due to the effect of those organs on the esophagus. Since all calculations are made using only bolus geometry, it is impossible to distinguish the effects of other organs outside the esophagus. Hence, we hypothesize that the lower values of ${\left(K/\theta \right)}_{\min}$ estimate the true stiffness of the esophageal walls, and the greater value of ${\left(K/\theta \right)}_{\min}$ near x=2 cm is likely a composite measure partly attributable to extrinsic compression. Close to the advancing peristaltic contraction, θ<1 , so ${\left(K/\theta \right)}_{\min}$ takes a greater value, and the esophagus seems to be locally stiffer. Moreover, it should be noted that we have not included the EGJ in Figure 13. This is because we did not define the problem with the tube law applied at the EGJ. After all, applying the tube law at the EGJ would not result in a unique solution. The mechanical properties of the esophageal walls have been estimated experimentally in several studies (Orvar et al., 1993; Patel and Rao, 1998; Kwiatek et al., 2011). In those studies, the esophagus was distended, and the cross-sectional area and the pressure developed inside were recorded. A straight line was fitted to quantify the linear relationship between cross-sectional area and pressure in an inactive esophagus. The slope of the line measured the quantity $A_{o}/K$ , which was in the range of 9.1–11.6 ${mm}^{2}/mmHg$ . Using the typical range of $A_{o}$ , as described in the study by Xia et al. (2009), i.e., 7−59 ${mm}^{2}$ , the stiffness of the esophageal walls was found to lie in the range of 0.6−6.5 mmHg. The effective stiffness, as shown in Figure 13, lay in the range of 1−7 mmHg, which is of the same order of magnitude as observed in the other studies.

**FIGURE 14:**
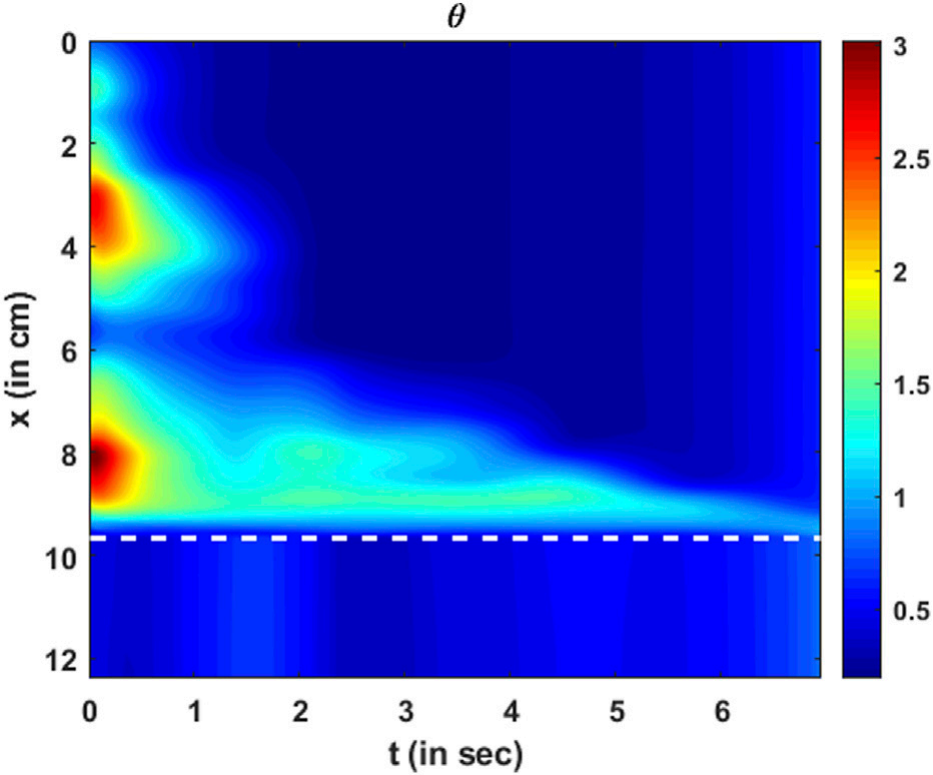
Variation in active relaxation as a function of x and t . The dashed line corresponds to the proximal end of the LES. Since the tube law was not specified at the LES, active relaxation is meaningful only in the esophageal body (above the dashed line).

The parameter θ quantifies the amount of active relaxation of the esophageal walls to facilitate distention and, consequently, decrease local intrabolus pressure and increase the flow rate. The variation in the active relaxation parameter θ is shown in Figure 14. As described in Eq. 31 and comparing Figures 10A; Figure 14, it is evident that the locations of the high values of θ match the locations of the high values of A , and similarly, lower values of θ match the lower values of A . It should be noted that θ quantifies the active relaxation in the esophageal body and not the LES. Comparing Figures 13, 14 shows that locations of greater stiffness correspond to locations of lower active relaxation and vice versa. Similar to ${\left(K/\theta \right)}_{\min}$ , as previously described, the impact of tissues and organs outside the esophagus also impacts the prediction of θ . Hence, the low value of θ near x=6 cm does not necessarily mean a lack of active relaxation but most likely the influence of structures outside the esophagus. Hence, we hypothesize that the greater values of active relaxation are closer to the actual active relaxation of the esophageal walls.

The MRI-MECH framework assumes constant values for the inactive cross-sectional area $A_{o}$ and gastric pressure to predict cross-sectional areas, velocity, and pressure as a function of x and t and, consequently, wall stiffness and active relaxation. The MRI-MECH’s sensitivity to using these constant values needs further discussion. The active relaxation parameter θ , as described in Eq 31, is not dependent on $A_{o}$ and, therefore, not sensitive to the magnitude of $A_{o}$ . The effective stiffness, on the other hand, is dependent on $A_{o}$ , as described in Eq 30. Thus, patients with a higher local inactive cross-sectional area than the global average $A_{o}$ will have lower effective stiffness. This observation is not unphysical since a higher inactive cross-sectional area allows more space for the bolus and effectively makes it appear more compliant. In summary, a 5%–10% variation in $A_{o}$ will lead to a 5%–10% variation in the predicted values of stiffness. It should be noted that flow velocity and pressure are not sensitive to the choice of $A_{o}$ . The estimate of stiffness is sensitive to the correct estimate of gastric pressure since dynamic pressure variations are negligible compared to the total pressure. Since the predicted estimate of stiffness, in this case, lies in the normal range (as expected since MRI was performed on a healthy volunteer), it is evident that the usage of 7 mmHg as gastric pressure is correct. The pressure predictions follow the same trend as the specified pressure boundary condition. For instance, if there is a 5%–10% variation in the estimated/assumed value of pressure, it would lead to a similar 5%–10% variation in the predicted values of pressure inside the esophageal body and, consequently, a 5%–10% variation in the predicted values of stiffness. It should be noted that active relaxation and flow velocity are not sensitive to the pressure boundary condition since they both depend only on cross-sectional areas.

We did not use any weight functions for the heterogeneous loss functions to calculate the total loss, as described in Eq. 21. However, there is a potential for increased accuracy with fine-tuned weights for the various loss functions, and that might vary with different patient data. The reason for not using different weights in this paper was to provide a baseline approach that can be easily implemented without major fine-tuning. Additionally, it should be noted that it is possible to divide the problem discussed in this paper into two parts for faster computation. In the esophagus proximal to the LES, we can calculate cross-sectional areas and velocities using Eqs 5, 6, respectively, followed by calculating pressure by numerically solving Equation 8. The pressure boundary condition at the distal end of the esophageal body (i.e., the proximal end of the LES) can be calculated using the tube law once $k_{r}$ is estimated. To infer physics inside the LES, the PINN approach remains the same as described in this paper, excluding everything proximal to the LES. The main purpose of including the entire domain in this paper is to provide a unified framework that calculates pressure variations inside the esophageal body, along with predicting missing information inside the LES.

An important aspect of the validation of this framework was to minimize all possible errors in predictions when compared with the measurements, specifically the variation of cross-sectional areas. Thus, our framework never predicts anything different from the measurements. Furthermore, an important characteristic of bolus transport is the physical transport of fluid through the esophagus, which must follow the laws of physics, specifically the mass and momentum conservation equations. Thus, low values of the residues for the mass and momentum conservation equations imply that MRI-MECH ensures that the physical laws are accurately followed. Finally, the estimate for esophageal wall stiffness for the normal subject who underwent the MRI procedure lay in the normal range, as reported in the studies by Orvar et al., (1993) and Kwiatek et al. (2011). This was another indirect approach to validate this framework.

### 3.1 Limitations

Although MRI-MECH provides valuable insights into the nature of transport and the mechanical state of the esophagus, it has limitations. Currently, manual segmentation of the bolus geometry is more accurate and reasonable for the low temporal resolution of the dynamic MRI, but it can become tedious with improved temporal resolution. Automatic segmentation using deep learning techniques might be helpful in that aspect, but itincreases the risk of inaccurate segmentation without a large training dataset. Bolus transport, as visualized in MRI, provides no information proximal to the bolus (a similar problem also occurs in fluoroscopy). Hence, MRI-MECH cannot predict anything meaningful proximal to the bolus. Thus, MRI-MECH cannot be used to estimate the contraction strength, for which other diagnostic techniques such as HRM or FLIP should be used. The esophageal wall properties and neurally activated relaxation were estimated solely through the bolus shape and movement. However, the bolus shape and movement depend not only on the esophageal walls but also on the impact of the organs surrounding the esophagus. This is a limitation of the MRI-MECH framework in predicting the state and functioning of the esophagus due to a lack of information about the impact of the surrounding organs. Finally, the prediction of intrabolus pressure and esophageal wall stiffness depends on the specification of the correct intragastric pressure. This becomes a limitation for MRI-MECH since the intragastric pressure is not known in MRI, so we used a reference value from the literature. Accurate measurement of the intragastric pressure through other diagnostic techniques, such as HRM, will increase the accuracy of the MRI-MECH predictions of intrabolus pressure and wall stiffness. The main focus of this study is to introduce a framework that can quantify the state and functioning of the esophagus through mechanics-based parameters for esophageal stiffness and active relaxation. This was accomplished with data from just one subject.

All the elements in the MRI-MECH framework would remain the same even if there were multiple subjects. Moreover, the physics-informed neural network utilized in this paper does not depend on data from many subjects as typical machine learning approaches do but rather is used as an optimizer focused on individual subject data. However, the success of any framework is determined by how efficiently it can be used on many subjects for clinical practicability. A detailed study of applying this framework to many subjects is beyond the scope of this work due to the current lack of availability of volunteers, and so, it is a limitation of this work. Finally, a true validation of this framework would be through comparison with other invasive methods used on the same subject simultaneously with MR imaging. Unfortunately, that was not available for the subject and remains a limitation of this work.

## 4 Conclusion

We presented a framework called MRI-MECH that uses dynamic MRI of swallowed fluid to quantitatively estimate the mechanical health of the esophagus. The bolus geometry, which tracks the inner cross-section of the esophagus, was extracted through manual segmentation of the MR image sequence and was used as input to the MRI-MECH framework. MRI-MECH modeled the esophagus as a one-dimensional flexible tube and used a physics-informed neural network to predict fluid velocity, intrabolus pressure, esophageal wall stiffness, and active relaxation. The PINN minimized a set of measurement losses to ensure that the predicted quantities matched the measured quantities and a set of residuals to ensure that the physics of the fluid flow problem was followed, specifically the mass and momentum conservation equation in one dimension. The LES cross-sectional area is very difficult to visualize in MRI because it is significantly smaller than the cross-sectional area at the esophageal body. In this regard, MRI-MECH enhances the capability of the dynamic MRI by calculating the LES cross-sectional area during esophageal emptying. We found that our predictions of the intrabolus pressure and the esophageal wall stiffness match those reported in other experimental studies. Additionally, we showed that the dynamic pressure variations that occur because of local acceleration/deceleration of the fluid were negligible compared to the total intrabolus pressure, whose main contribution was the elastic deformation of the esophageal walls. The mechanics-based analysis with detailed three-dimensional visualization of the bolus in MRI leads to significantly better prediction of the state of the esophagus than two-dimensional X-ray imaging techniques such as esophagram and fluoroscopy and can be easily extended to other medical imaging techniques such as computerized tomography (CT). Thus, MRI-MECH provides a new direction in mechanics-based non-invasive diagnostics that can potentially lead to improved clinical diagnosis.

## Data Availability

The raw data supporting the conclusion of this article will be made available by the authors, without undue reservation.
