# Supplementary material for: MRI-MECH: mechanics-informed MRI to estimate esophageal health
Source: Front Physiol. 2023 Jun 9;14:1195067. doi: 10.3389/fphys.2023.1195067 (PMC10289887; doi:10.3389/fphys.2023.1195067)
Supplement: Supplementary file 1 [file Table1.DOCX]

Supplementary Material

MRI-MECH: Mechanics-informed MRI to estimate esophageal health

Sourav Halder, Ethan M. Johnson, Jun Yamasaki, Peter J. Kahrilas, Michael Markl, John E. Pandolfino, Neelesh A. Patankar^*^

*** Correspondence:** Neelesh A. Patankar: n-patankar@northwestern.edu

# MRI segmentation using deep learning

In this work, we also demonstrate the use of a convolutional neural network (CNN) called a 3D U-Net to perform semantic segmentation based on a limited number of 2D slices of the 3D MRI image. The 3D U-Net is built on the U-Net, which is composed of a contracting encoder path and an expansive decoder path. A key differentiating feature of the 3D U-Net is that instead of operating on the 2D images that the U-Net expects as input, the former operates on volumetric (3D) data. Automatic segmentation using the 3D U-Net was viable due to the repetitiveness of the structures and variations of the input images, especially for volumetric biomedical data.

The network architecture used was the same as the 3D U-Net, with the only change being that the number of channels is 1, instead of 3. Batch normalization was performed after each convolutional layer to prevent overfitting. The network had a total of 16,324,929 parameters, of which 4,672 were non-trainable and 16,320,257 were trained.

The 3D U-Net was trained using 5 training sets, with each consisting of an image and a mask. The original MRI scans are composed of folders for each time instant, which contain a series of 64 .dcm files representing each slice. MRIcron, a NIfTI image viewer that contains the DICOM to NIfTI converter dcm2nii, was used to convert these raw images into .nii files to be loaded into the model. To prepare the training masks, we used ITK-SNAP to perform segmentation via manual delineation, where voxels in the images were given a label of 1 if they were determined to be part of a bolus, and 0 if they were not. For the benchmark case, the images were manually segmented with intervals of 2, 4, and 2 slices respectively in the x, y, and z directions. In order to determine the effects of the sparsity of the annotation on the training of the model as well as the accuracy of the prediction, manual segmentations were made on the same set of images using the same method, except with intervals in the x, y, and z directions of (4, 8, 4), (4, 8, 8), in addition to a training set with only one segmentation slice in each direction to represent a more extreme case of sparse annotation. Another image/mask set was used for validation during training, and the 7th set was for testing to compare prediction accuracy among the different cases. The amount of data used in the training of the model is adequate, as in many biomedical volumetric image classification situations, only two images are required to attain reasonable accuracy, along with a weighted loss function and data augmentation.

The original images and masks were of size 160 × 160 × 64, but we implemented a cropping procedure along the x-direction such that the eventual inputs to the neural network were of size 160×80×64. This was done in an endeavor to reduce the number of voxels in the input image and thereby reduce processing time, whilst still retaining the area of focus – the esophagus and bolus. Data augmentation was also implemented using techniques such as Gaussian blur, image sharpening, random variation of image brightness, contrast normalization, and elastic deformation.

For our segmentation problem, we used the weighted Sorensen Dice Coefficient to measure the similarity between the predictions and the training set. The weighted dice coefficient loss was used as the loss function during training.

Several hyperparameter tuning trials were conducted to find the optimal hyperparameters and conditions for training the model. Through experimentation, we found that training for 1000 epochs with an initial learning rate of 10−2 using a learning rate scheduler to decrease the learning rate by a factor of 3 every 250 epochs proved to be the most reliable. The training was conducted using Keras, a high-level neural network API of TensorFlow, and the optimization algorithm used was stochastic gradient descent (SGD). A maximum weighted dice coefficient of 0.8575 was attained at epoch 282 during training. Figure I shows the learning curves and the weighted dice coefficient. The predictions were converted to their binary forms with voxels with values greater than 0.05 classified as 1 (bolus), and those equal to or below 0.05 are set to 0 (background). Figure II shows the predictions at some slices in the 3D MRI.

Retaining the same hyperparameters, we also ran trials with different levels of annotation sparsity. Let us denote the number of slices in the x, y, and z directions as (Nx, Ny, Nz). The benchmark trial had (2,4,2), and saw a final validation weighted dice coefficient of 0.7679. The final weighted dice coefficient for the (4,8,8), (4,8,4), and (1,1,1) cases were respectively 0.4608, 0.5289, and 0.3655. Thus, in this trial, we found that the benchmark case of (2,4,2) yielded the highest accuracy.

Ultimately, the training of a 3D U-Net and its application for the ascertainment of the location and shape of the bolus was to evaluate the potential of using the output data for MRI-MECH. In this regard, there may be room for further work, particularly in the post-processing of the prediction since more accuracy in the prediction is desirable for mechanics-based simulations.

# Supplementary Figures


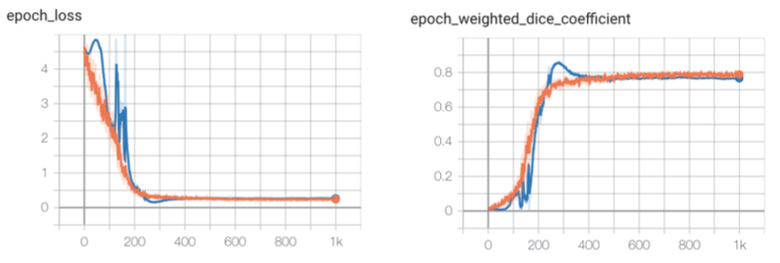


Supplementary Figure 1. Loss and weighted dice coefficient during training for the benchmark case. The curve for the training set is in orange, and blue represents the validation set.


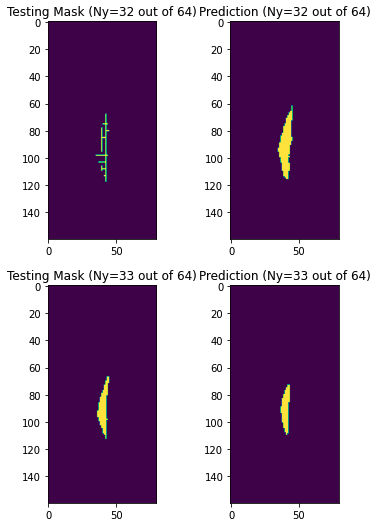


Supplementary Figure 2: Side-by-side comparison of the testing mask (annotated manually) vs. the prediction for the benchmark case for some sample y locations.
